# Integrating a family-focused approach into child obesity prevention: Rationale and design for the My Parenting SOS study randomized control trial

**DOI:** 10.1186/1471-2458-11-431

**Published:** 2011-06-05

**Authors:** Dianne S Ward, Amber E Vaughn, Kant I Bangdiwala, Marci Campbell, Deborah J Jones, Abigail T Panter, June Stevens

**Affiliations:** 1 Department of Nutrition in the Gillings School of Global Public Health, University of North Carolina at Chapel Hill, USA; 2 Center for Health Promotion and Disease Prevention, University of North Carolina at Chapel Hill, USA; 3 Department of Biostatistics in the Gillings School of Global Public Health, University of North Carolina at Chapel Hill, USA; 4 Department of Psychology, University of North Carolina at Chapel Hill, USA

## Abstract

**Background:**

More than 20% of US children ages 2-5 yrs are classified as overweight or obese. Parents greatly influence the behaviors their children adopt, including those which impact weight (e.g., diet and physical activity). Unfortunately, parents often fail to recognize the risk for excess weight gain in young children, and may not be motivated to modify behavior. Research is needed to explore intervention strategies that engage families with young children and motivate parents to adopt behaviors that will foster healthy weight development.

**Methods:**

This study tests the efficacy of the 35-week My Parenting SOS intervention. The intervention consists of 12 sessions: initial sessions focus on general parenting skills (stress management, effective parenting styles, child behavior management, coparenting, and time management) and later sessions apply these skills to promote healthier eating and physical activity habits. The primary outcome is change in child percent body fat. Secondary measures assess parent and child dietary intake (three 24-hr recalls) and physical activity (accelerometry), general parenting style and practices, nutrition- and activity-related parenting practices, and parent motivation to adopt healthier practices.

**Discussion:**

Testing of these new approaches contributes to our understanding of how general and weight-specific parenting practices influence child weight, and whether or not they can be changed to promote healthy weight trajectories.

**Trial Registration:**

ClinicalTrials.gov: NCT00998348

## Background

Preventing obesity in children requires intervening early, before a child becomes overweight and an unhealthy weight gain trajectory is established. Current estimates from the US indicate that even among the youngest children, those aged 2-5 years, more than 20% are already overweight or obese [1]. The literature consistently demonstrates that rapid growth and excess weight during childhood increases the risk of obesity later in life [2]. Excess weight also increases a child's risk for many adverse outcomes related to their short and long term physical and mental health, including high blood pressure, diabetes, elevated cholesterol, fatty liver disease, asthma, depressive symptoms, anxiety, low self-esteem, low body image, and mood and conduct disorders [3-5].

Despite the alarming prevalence of overweight in children and the health risks associated with excess weight, prevention of child obesity is not a seen as a priority for many parents. This remains the case in spite of widespread understanding among professionals that obesity prevention efforts must engage parents who are the key gatekeepers that shape the social and physical environment of the home, and, as a result, influence their child's diet and physical activity behaviors [6-8]. Multiple studies have demonstrated that most parents are unable to recognize when their child is overweight, especially younger children [9-12]. Even when parents do appreciate the importance of good nutrition and physical activity, their day-to-day practices are likely to be determined by more acute concerns regarding child health and behavior, rather than the more sustained attention and effort necessary to prevent obesity [13].

Given the critical need to educate parents about their role as gatekeepers in the prevention of obesity, it is surprising that relatively little empirical attention has been devoted to intervening in families with preschool-age children before unhealthy nutrition and physical activity patterns have been entrenched [14]. Lack of parent intervention models may be due in part to the many challenges associated with this approach. Creating a home environment that supports healthy weight development is not as simple as providing parents with information about nutrition and physical activity recommendations. Parents must be convinced to make obesity prevention a priority despite the multiple, often competing demands on their time and resources. The purpose of this manuscript is to describe the program and research design of a parent-focused intervention for healthy weight development in young children called *Parenting SOS: Strategies of Success for Raising Strong and Healthy Children*. Descriptions will follow the Consolidated Standards of Reporting Trials (CONSORT) reporting guidelines.

## Theoretical Underpinnings and Conceptual Model

In order to create an intervention for parents of preschool-age children that promotes healthy weight gain, it was important first to understand the family context and how it influences child weight and weight-related behaviors, as well as how to motivate behavior change in both parents and children. Darling and Steinberg's Integrative Model of Parenting provides a conceptual model for understanding the family system and how parent variables (values, parenting style, and parenting practices) affect child behavior [15]. This model suggests that parents' values and goals, related to their child learning specific skills or behaviors (e.g., living up to their academic potential, listening to and obeying rules and instructions, eating healthy and being active) or developing certain character qualities (being confident, resilient, empathetic, balanced), will influence parent practices and style. Parent practices are the specific strategies or behaviors parents use to help their child achieve certain goals; and practices have a direct effect on child outcomes. Style, on the other hand, is thought to moderate the influence that parenting practices have on child behavior. Style affects the parents' ability to socialize their child and the effectiveness of their parenting practices.

Ryan and Deci's Self-Determination Theory provides a framework for understanding motivations, and suggests approaches for building intrinsic motivation to adopt healthier behaviors [16]. This theory suggests that self-motivation can be enhanced via fulfillment of three innate psychological needs - competence, relatedness, and autonomy. Competence can be enhanced through positive feedback, communications, and rewards that are supportive but also challenging. Relatedness, or the need to feel connected with others, can be enhanced by having behaviors prompted, modeled, or valued by significant others. Autonomy can be enhanced through providing choices, acknowledging feedings, and offering opportunities for self-direction.

**Figure 1:**
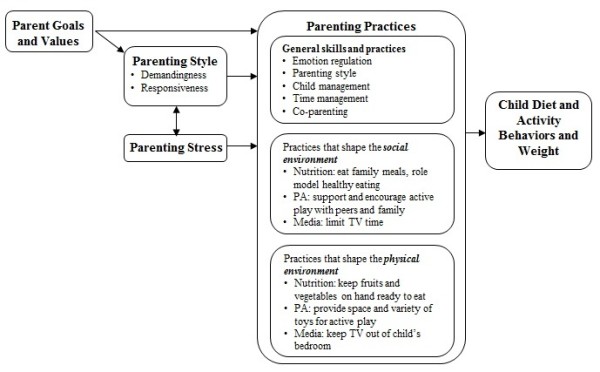
**Conceptual model**. The conceptual model for the Parenting SOS program is based on Darling and Steinberg's Integrative Model of Parenting Ryan and Deci's Self-Determination Theory, and incorporates parent goals and values, parenting style and stress, and parenting practices, which could influence child diet and activity behaviors as well as child weight.

These two theoretical models serve as the basis for the current conceptual model for the parenting for healthy weight intervention described below (Figure 1). This model and the resulting program were developed with the belief that general parenting is the gateway activity for parents to develop appropriate parenting skills for their children's healthy eating and activity patterns.

## Methods

**Figure 2:**
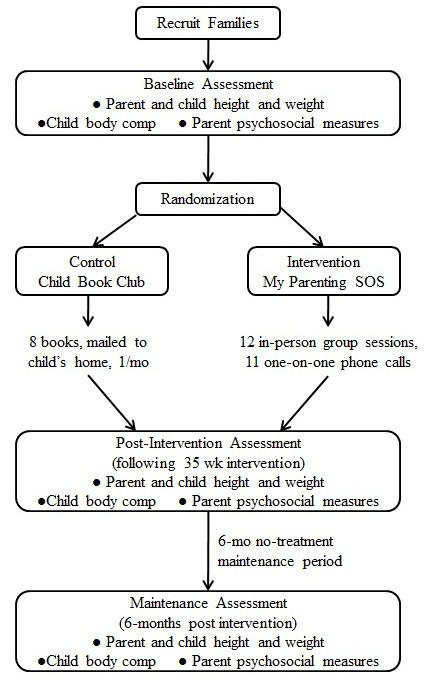
**Intervention model**. Participants are recruited into the study and complete a baseline assessment before randomization. After either the 8-month My Parenting SOS intervention or child book club, participants complete a post-intervention assessment. The maintenance assessment are completed after a 6-month no-treatment period.

This study uses a randomized control design to test the efficacy of *My Parenting SOS*, a 35-week intervention for families with preschool-age children, designed to promote parenting practices that lead to healthy eating and activity behaviors in children. All study procedures have been reviewed and approved by the Institutional Review Board at the University of North Carolina at Chapel Hill (IRB Study # 08-0354). An overview of study design is depicted in Figure 2.

### Study Hypotheses

The study's primary hypothesis is that, by the end of the 35-week intervention, children in the intervention arm will have a lower mean change in percent body fat compared to children in the control arm. Secondary hypotheses include: that, in comparison to children in the control arm, children in the intervention arm will: (1) improve the quality of their dietary intake, (2) increase their physical activity, and (3) maintain a lower mean percent body fat following a 6-month, no-treatment maintenance period. We also hypothesize that parents in the intervention arm, in comparison to those in the control arm, will: (1) improve the quality of their dietary intake, (2) increase their physical activity, and (3) improve their general parent behaviors (i.e., general parenting style and practice) as well as domain-specific practices around feeding and activity. In addition, we will examine the mediating effects of parent motivation, general parenting style and practices, and parenting for healthy weight on the impact of the 35-week parenting intervention on child diet and physical activity behaviors and body fat; although due to the exploratory nature of this aim no hypotheses are offered.

### Participants and Recruitment

Families with preschool-age children are being recruited in three waves using a variety of community resources including child care centers, direct mailings, community bulletin boards, listservs, and newspaper community announcements. The recruitment plan incorporates strategies to engage families from diverse racial, ethnic and socioeconomic backgrounds. For example, recruitment efforts are focused on counties located in central North Carolina with a large percentage of minority residents. Additionally, child care centers that accept subsidies are specifically targeted to engage low-income families.

Interested families are screened by phone. Eligibility criteria include having at least one child between the ages of 2 and 5 years old, at least one parent with a body mass index (BMI) greater than 25 (based on self-reported height and weight), willingness to participate in measures and intervention activities, and ability to speak English or comprehend standard age-level materials. Signed consent is then obtained from all participants when they arrive for baseline data collection. Parents sign consent for their own participation and that of their child.

### Power Calculation

The study is powered on its primary outcome: mean change difference in child percent body fat, estimated from the Dezenberg et al. [17] formula between intervention and control children. The power calculation assumed an absolute change difference in percent body fat of 2% at the 35-week post-intervention point. The power calculation determined that 280 families needed to be recruited and randomized to provide at least 80% power, assuming a two-sided test at the 5% level, and a retention rate of 80% at the post-intervention assessment.

### Randomization

For each wave, participants are first stratified by income (household income more or less than $50,000) and county. Household income of $50,000 represents the approximate median household income for the counties targeted for recruitment and was therefore selected as the cut point for income stratification. For each wave and county combination, two randomization tables (one for each income level) are created using SAS 9.2 (SAS Institute, Cary NC) with a randomization allocation ratio of 1:1 and a block size of 2 to ensure balance between study arms across all strata. These randomization tables are then used to create sets of sequentially ordered cards that are sealed so that the condition assignment (control vs. intervention) appearing inside is not visible.

Participants have the opportunity to select a card and be randomized upon arrival to a kick-off event. For each wave, three to four kick-off events are offered. At the beginning of each event, cards from each of 2 stacks are placed into either pail "A" or "B", corresponding to income strata. The number of cards in the pail always exceeds the expected number of participants from each income stratum expected to attend the event. Cards in each pail are shuffled and as participants arrive, staff instruct participants to select one card from the appropriate pail. Cards remaining from the first event are left in the pails and used for the next event, with additional cards added as needed. Stratification procedures are not communicated to the participants.

At these kick-off events, families randomized into the intervention group immediately start their first session, while those randomized into the control group have a short child entertainment program (e.g., book reading, puppet show). Participants must complete baseline measures and attend one of these kick-off events in order to be randomized into the intervention or control group, and continue their participation in the study. This creates a run-in period and helps minimize loss to follow-up.

### Intervention

The *My Parenting SOS *program was developed by a team of investigators with backgrounds in nutrition, physical activity, child obesity prevention, child and family psychology, and health communication (unpublished manual, *My Parenting SOS Leader's Guide*); and builds off of multiple phases of pilot work which are summarized briefly in Table 1. Lessons gleaned from this work affirmed the need to target parents with preschool-age children, and to integrate general parenting styles and specific food- and activity-related parenting practices.

**Table 1:** Formative and pilot work

Date	Activity	Objectives
June 2004 - Jan 2005	Focus groups with mothers of overweight children	Identify culturally specific child management concerns and behavioral intervention needs of parents and/or caregivers, and their preferences for intervention channels and modalities [47]

Mar - May 2005	Focus groups with AA mothers	Explore barriers to healthy nutrition and activity practices at home, collect impressions regarding a parenting for healthy weight intervention, and assess preferences regarding intervention delivery

Sept - Oct 2006	Pilot of 4-part workshop series with parents from 1 local child care center	Examine the acceptability of a parenting for healthy weight workshop series and evaluate appropriateness of potential measures

Oct - Dec 2006	Pilot of 3-part workshop series with parents from 6 local child care centers	Examine the feasibility of workshop delivery by trained facilitator (with a social work background) provided at the child care center

The program is delivered through 12 in-person group meetings and 11 tailored phone calls across 35 weeks. The group meetings include separate programs for parents and their children.

#### Parent Program

The parent meetings are facilitated discussions about select parenting skills, and initial sessions address general parenting topics including: parenting style, child behavior management, stress management, coparenting relationships, and family routines. Later sessions show parents how to apply these skills to promote healthy nutrition and physical activity behaviors in their children. Table 2 summarizes the key parenting and health messages in each of the sessions. Parents receive personal counseling calls from the interventionist between each of the sessions. This call is used to discuss successes and challenges encountered as the parent tries to implement the new skills from the previous session. Motivational interviewing informed techniques are used by the facilitator during these calls, working to encourage participants to identify their own challenges and options for overcoming those obstacles.

**Table 2:** Intervention overview

Sessions	Key parenting messages	Nutrition/PA messages/activities
Stress management	• Identification of common parenting stressors • Effect of stress on parenting • Strategies for managing stress	• Examples of ineffective coping strategy = using food/alcohol as source of comfort • Examples of effective coping strategies = taking care of yourself - eating a healthy diet and getting regular exercise

Parenting style	• Introduction of 4 major parenting styles (authoritarian, authoritative, indulgent, uninvolved) • Importance of balancing warmth and support with discipline	• Discuss strategies that parents with different styles might use to get their child to eat fruits and vegetables, and the pros and cons of each

Child management (2 sessions)	• Strategies for managing child behavior (attending, reinforcement and rewarding, effortful ignoring, setting clear rules and limits, choices, consequences, time out)	• Use of attending and verbal reward to encourage intake of healthy foods • Alternatives to using food as a reward or bribe to control child's behavior

Emotion regulation	• Improve understanding of how parents contribute to their child's emotional development through role modeling, their own response to their emotions, and their parenting behaviors	• Re-emphasize examples of ineffective and effective coping strategies and that children will learn these from their parents' example • Food should not be a substitute for love and affection

Coparenting	• Strategies for strengthening the communication between coparents so that they can present a united front	• Encouraging parents to present a united front when it comes to healthy habits

Family routines	• Introduction to creating routines that can improve efficiency and predictability at home	• Planning time for healthy meals should be part of morning and evening routines

Nutrition and feeding practices (2 sessions)	• Routines can help parents find the time to plan, prepare, and eat meals as a family • Use of the authoritative parenting style and choices can help parents encourage healthy eating • Attending and verbal praise is a way to reinforce children's healthy choices	• Introduction to the food guide pyramid • Identification of go, slow, and whoa foods • Appropriate portion sizes • Family meals • Overcoming picky eating behaviors

Physical activity and activity practices (2 sessions)	• Planning ahead can help parents make the time for family activity • Rethinking house rules to accommodate active play • Using rules and limits to manage time children spend in sedentary activities	• Introduction to physical activity guidelines for adults and children • Examples of different levels of activity • Age-appropriate physical activities • Being active as a family • Managing media time

Sleep habits	• Creating bedtime routines can help parents get children to bed with fewer battles • Incorporating quality time between parent and child as part of the bedtime routine • Using child management skills to help limit/discourage the child from repeatedly getting up during the night	• Sleep recommendations for children and adults • Adverse health outcomes associated with insufficient sleep

#### Child Program

The child program grew out of our pilot work where parents indicated that a program beneficial to their child, rather than just offering child care, would provide additional motivation for their own attendance. The child program is designed to reinforce the parent program and prepare children for changes they would be seeing at home related to new parenting practices and the encouragement to eat healthy and be active. Each session follows a set routine that incorporates active play time, a family-style meal, a music and movement game, an enrichment activity, a new food taste testing, and story time. Specific games, activities, and books are selected to reinforce that week's key lesson. Lesson topics include: rules and limits, emotions, working as a family and family routines, and healthy lifestyles.

### Control Condition

Families randomized into the control group are enrolled into a child's book club. The child entertainment program offered at kick-off events is intended to help launch the book club program and is generally conducted by local librarians. Families receive one book each month, mailed directly to their home, for the duration of the 35-week intervention period. Book titles for this arm were identified with the assistance of a child's librarian. Books related to health, nutrition, and physical activity were purposefully avoided when selecting titles. Mailing of books directly to participants' homes also helps monitor for changes of address among control families.

### Outcomes and Measures

Outcomes are assessed at three time points: baseline, post-intervention, and maintenance (6-month post). Measurement events are scheduled at convenient community locations so that participating families can come in and complete measurements. Locations allow for multiple stations including check-in, anthropometric measures, parent psychosocial surveys, accelerometry, and check-out. Locations also provide space for child activities to keep children entertained while parents complete surveys.

#### Primary Outcome

The primary outcome is mean change difference in child percent body fat between baseline and post-intervention. Percent body fat is calculated using the formula of Dezenberg et al.[17] which incorporates weight, triceps skinfold, gender, and race/ethnicity. Collection of anthropometric data is described in detail below; sex is captured by the technician during measurement; and race/ethnicity is reported by the parent. The Dezenberg equation has been validated in 4-11 yr old Caucasian and African American children and found to be highly predictive of body fat mass measured by DEXA (R^2 ^= 0.95, Model SEE = 0.50)[17].

#### Secondary Outcomes

##### Anthropometry

Standing height and weight are measured by a trained technician on both children and parents. Height is measured to the nearest 1/8 inch with a Shorr or Seca infant/child/adult measuring board (Shorr Productions, Olney, MD; Seca Corporation, Columbia, MD); and weight is measured to the nearest 0.1 lb with a Seca model 770 portable electronic scale (Seca Corporation, Columbia, MD). Additional anthropometric measures taken on children are triceps and subscapular skinfold thickness and waist circumference. Skinfold thicknesses are measured to the nearest 1.0 mm using Lange calipers (Beta Technology, Inc. Cambridge, MD); and waist circumference is measured to the nearest 0.1 cm using a Gulick II measuring tape. These measures are also used to calculate child and parent BMI, child BMI z-score, and child sum of two skinfolds.

##### Diet

Parent and child diet are assessed using three days (2 weekdays and 1 weekend day) of unannounced 24-hour dietary recalls collected over a 4-week period. All recalls are conducted under the direction of the UNC Nutrition Epidemiology Core (CNRU, NIH DK056350) using traditional multi-pass procedures which provide cues for portion size, use of condiments, etc. [18-20]. All recalls are conducted with parents who are asked to recall what they ate the previous day and what their child ate while in their care. The NDS-derived analyses conducted on three 24-hr dietary recalls allows for analysis of intakes of energy, saturated fat, servings of fruits, vegetables, and whole grains, and intake of sugar-sweetened beverages.

##### Physical Activity

Parent and child physical activity are assessed with ActiGraph GT3X accelerometers (ActiGraph, LLC, Fort Walton Beach, FL). Parent monitors are programmed to collect data in 1-min epochs, while child monitors use a 15-sec epoch to capture their variable movement patterns. Monitors are worn over the right hip, for seven consecutive days, during waking hours, except when in water. Data are reduced using the adapted SAS code used in NHANES, and appropriate cut-points are applied to determine time spent in sedentary, light, moderate, and vigorous physical activity. We will use the current NHANES accelerometer cut-points used for parent data [21], while cut-points recently developed by Evenson et al. for 3-5 yr old children are used for child data [22]. The ActiGraph has been found to be effective for measuring both physical activity and inactivity in preschool-age children [23-25].

##### General Parenting Practices

Existing scales are also employed to measure parent practices addressed as part of the intervention, including: (1) the Alabama Parenting Questionnaire - Preschool Revision (APQ-PR), which includes subscales for positive parenting, inconsistent parenting, and punitive parenting [26]; (2) the Difficulties in Emotion Regulation Scale (DERS), which assesses parent ability to manage their own emotional responses and includes subscales for non-acceptance of emotional responses, difficulties engaging in goal-directed behavior, impulse control difficulties, lack of emotional awareness, limited access to emotion regulation strategies, and lack of emotional clarity [27]; (3) the Coping with Children's Negative Emotions Scale (CCNES), which assesses how parents react to children's negative affect in distressful situations and includes subscales for distress reactions, punitive reactions, expressive encouragement, emotion-focused reactions, problem-focused reactions, and minimization reactions [28,29]; (4) the Confusion, Hubbub, and Order Scale (CHAOS), which assesses parental time management skills (e.g., use of routines) [30]; and (5) the Parenting Convergence Scale (PCS), which assesses the degree to which parents work together to tackle parenting responsibilities and includes subscales for communication, conflict, and support [31,32].

##### Parent Feeding Practices

Parent feeding style and behaviors are assessed using a combination of items from existing instruments including: (1) the Caregiver's Feeding Style Questionnaire (CFSQ), which assesses two dimensions of parent feeding - demandingness and responsiveness) [33]; (2) the Parental Feeding Style Questionnaire (PFSQ), which includes subscales for emotional feeding, instrumental feeding, prompting/encouragement to eat, and control over eating [34]; and (3) nine subscales from the Comprehensive Feeding Practices Questionnaire (CFPQ), specifically those for child control, child involvement, parent modeling of healthy eating, parent monitoring of child intake, parental pressure to eat, parent restriction for health, parent restriction for weight control, encouraging of balance and variety, and teaching about nutrition [35].

##### Parent Physical Activity-Related Practices

Measures of parent practices specific to physical activity are not well developed; therefore, a tool was developed by investigators based on an extensive literature review (unpublished data) and using items and scales taken or modified from existing instruments [36]. Items assess constructs such as indoor/outdoor rules, use of physical activity as a reward or punishment, monitoring, control, support, parent role-modeling, family activity, praise, encouragement, education, and use of screen time for distraction.

##### Parent Motivation to Change Practices

Given the inclusion of the Self-Determination Theory Framework in our conceptual model, measures of intrinsic/autonomous motivation to change behavior are also assessed. Autonomous motivation to change is assessed with the Treatment Self-Regulation Questionnaires (TSRQ), which measures controlling vs. autonomous reasons an individual has for adopting certain healthy behaviors [37]. The original measure was modified for this study to ask parents why they would adopt more positive parenting practices. Parents' feelings of competence are assessed using three separate measures: one focusing on feelings of competency related to their role as a parent, and the last two focusing on feelings of competency specific to helping their child meet dietary and physical activity recommendations. Additional measures include the Parenting Sense of Confidence (PSOC) scale, which assesses parents' self-efficacy (competence, problem-solving ability, and capability) and satisfaction (affective dimension reflecting frustration, anxiety, and motivation) related to their role as a parent [38]; as well as the Parenting Self-Efficacy for Children's Healthy Weight Behaviors (PSE-CHWB), an instrument developed by investigators to assess parents' self-efficacy for adopting parenting behaviors that will help their children meet dietary and physical activity guidelines and promote healthy weight gain. Additionally, parent feelings of relatedness (i.e., attachment, social integration, reassurance of worth, reliable alliance, guidance, and opportunity for nurturance) are assessed using a modified version of the Social Provisions Scale (SPS) [39,40]. Items were adapted for this study to ask specifically about social support for general parenting.

##### Other variables

Several other variables thought to be related to the primary constructs of interest are assessed, including parenting stress (Parenting Stress Index [41]), parent depressive symptoms (Center for Epidemiologic Studies Depression Scale [42]), and child behavior (Eyberg's Child Behavior Inventory [43]), but will not be described in detail in this paper.

### Evaluation

Descriptive statistics will be used to compare the demographic characteristics of intervention and control arm participants at baseline - medians for continuous variables and proportions for categorical variables. All statistical tests and exploratory and confirmatory factor analyses on the physical and parenting and child psychological measures will be performed with either SAS 9.2 (SAS Institute, Cary NC) and MPlus [44]. Statistical tests will be conducted using a two-tailed test with an alpha value set at 0.05. Intervention implementation will be described using process evaluation data based on Glasgow's RE-AIM framework [45] and Kirkpatrick's Training Evaluation Model [46].

An intention-to-treat approach will be used for the primary hypothesis: the comparison of the mean change difference in child percent body fat from baseline to post-intervention between intervention and control children. We expect that our intervention will be effective in producing an absolute difference in change in percent body fat of 2%-3% between the two arms at post-intervention (35 weeks), but power was calculated based on the less optimistic 2% and with a conservative two-sided test of significance.

## Discussion

The Parenting SOS program offers an innovative approach to addressing healthy weight development in families with preschool-age children. The intervention is based on considerable formative and pilot research, and addresses general parenting as the gateway activity for parents to develop appropriate parenting skills for their children's healthy eating and activity patterns. Responding to our formative data, we developed a companion child program that offers our families support in making changes to current lifestyle practices. In addition to the innovative intervention content, the Parenting SOS program is theory-driven based on Self-Determination and Social Cognitive theories.

Because of the pioneering nature of this study, we include numerous measures of the social, environmental, and behavioral components of family life in anticipation to increase our understanding of the factors that moderate and mediate healthy weight parenting behaviors and, ultimately, child body fat development.

## Competing interests

The authors declare that they have no competing interests.

## Authors' contributions

DW is the principal investigator; she led the conception and design of the study, development of the intervention, selection of primary and secondary measures, and development of the manuscript. AV provides project management and coordination; she has contributed to the development of the intervention, selection of measures, and assisted with the development of the initial manuscript draft and incorporated revisions from co-investigators. KB provides biostatistical oversight for the study; he has contributed to the study design, calculation of power, development of randomization strategies, and has critically reviewed and edited the current paper. MC contributed to the development of the intervention and behavior change strategies, and reviewed/approved the manuscript. DJ contributed to the development of the intervention, identification of measures related to general parenting style and practices, and participated in the writing and editing of the current manuscript. AP provided guidance on data analysis strategies particularly plans for meditational analysis, assistance in selection of appropriate measures, and critically reviewed and edited the current paper. JS contributed to the development of the study design, selection of measures and randomization strategies, provided guidance on the scope of the manuscript, and has critically reviewed and edited the current paper. All authors have reviewed and approved the current version of the manuscript.

## Pre-publication history

The pre-publication history for this paper can be accessed here:

http://www.biomedcentral.com/1471-2458/11/431/prepub
